# Development and validation of a risk score (CHANGE) for cognitive impairment after ischemic stroke

**DOI:** 10.1038/s41598-017-12755-z

**Published:** 2017-09-29

**Authors:** Russell J. Chander, Bonnie Y. K. Lam, Xuling Lin, Aloysius Y. T. Ng, Adrian P. L. Wong, Vincent C. T. Mok, Nagaendran Kandiah

**Affiliations:** 1 Department of Neurology, National Neuroscience Institute, 11 Jalan Tan Tock Seng, Singapore, 308433 Singapore; 2 Department of Medicine and Therapeutics, The Chinese University of Hong Kong, 9/F., Lui Che Woo Clinical Sciences Building, Prince of Wales Hospital, Shatin, N.T. Hong Kong; 3 Duke-NUS Medical School, 8 College Road, Singapore, 169857 Singapore

## Abstract

Post-stroke cognitive impairment (PSCI) warrants early detection and management. We sought to develop a risk score for screening patients at bedside for risk of delayed PSCI. Ischemic stroke survivors with PSCI and no cognitive impairments (NCI) 3–6 months post-stroke were studied to identify candidate variables predictive of PSCI. These variables were used to develop a risk score using regression models. The score, and the best identified clinical cutoff point, underwent development, stability testing, and internal and external validation in three independent cohorts from Singapore and Hong Kong. Across 1,088 subjects, the risk score, dubbed CHANGE, had areas under the receiver operating characteristics curve (AUROC) from 0.74 to 0.82 in detecting significant risk for PSCI, and had predicted values following actual prevalence. In validation data 3–6 and 12–18 months post-stroke, subjects with low, medium, and high scores had PSCI prevalence of 7–23%, 25–58%, and 67–82%. CHANGE was effective in screening ischemic stroke survivors for significant risk of developing PSCI up to 18 months post-stroke. CHANGE used readily available and reliable clinical data, and may be useful in identifying at-risk patients for PSCI.

## Introduction

Survivors of ischemic strokes may encounter complications that can affect their functional outcomes and recovery process^1^. Post-stroke cognitive impairment (PSCI) refers to the development of cognitive deficits after a clinical stroke with no evidence of any major cognitive decline before the stroke, and is one of the leading factors contributing to this loss of independence^2^. The prevalence of PSCI in the existing literature ranges from 22%^3^ to 58%^4^, and varied depending on cohort, criteria used to define PSCI, and post-stroke duration studied, which can range anywhere from 3 months to 3 years^5,6^. PSCI can be insidious, affecting stroke survivors’ ability to live independently even after they have recovered physically^5^. PSCI can also increase the risk of other complications, such as recurrent strokes^2,7^, further cognitive decline especially with recurrent strokes^8^, and increased mortality^2,9^.

Due to the delayed onset of PSCI and possibility of cognitive improvement with time due to improved cerebral perfusion, cognitive performance may fluctuate between the subacute and chronic stages of stroke^10,11^. Hence, cognitive status post-acute stroke can be unreliable in predicting long-term cognitive status^10^. Nevertheless, other PSCI risk factors present in the subacute phase of stroke have been identified, including age^11,12^, education^11,12^, vascular risk factors^4,13^, extent of ischemia^4,11,12^, cerebral atrophy^12^, and ApoE expression^14^. While there is no shortage of PSCI risk factors for clinicians to consider, establishing the most salient of these factors and organizing them in a structured manner would make them more useful to clinicians. Furthermore, the development of a risk score for PSCI specifically would carry more content validity for subjects of this specific risk profile than adapting a stroke risk score or stroke-free dementia risk score to predict PSCI.

The recently developed SIGNAL_2_ scale^15^ for predicting PSCI was shown to be sensitive in identifying stroke survivors at high risk of PSCI at 3–6 months and 12–18 months post-stroke. However, several limitations needed to be addressed, including: 1) the need for external validation in an independent cohort, 2) the need for replacing variables not available in many clinical stroke centers, such as magnetic resonance angiography (MRA) or CT angiography (CTA), and 3) the need to incorporate different quantifying methods for cerebral atrophy or white matter disease. The objective of this current study was to develop a new clinical risk score for identifying mild ischemic stroke survivors at significant risk of PSCI that can address the issues of previous methods, and to validate the new score using both internal and external cohorts.

## Methods

### Risk score development

Three separate datasets were used in the development and validation of the risk scale. For the development cohort, an existing retrospective dataset consisting of acute ischemic stroke patients who attended a tertiary stroke center in Singapore between January 1, 2008 and December 31, 2012 was used. All ischemic strokes were confirmed by acute infarcts visualized in MRI along with the corresponding clinical symptoms. In order to focus on non-disabling strokes, only subjects with a modified Rankin score (mRS)^16^ of ≤2 at the time of discharge from the acute stroke inpatient stay were included. Pre-stroke cognitive impairment was defined by a score of ≥3.6 on the Informant Questionnaire of Cognitive Decline in the Elderly (IQCODE)^17^ administered to subjects’ relatives. Here, the IQCODE was modified to identify symptoms of cognitive decline within a period of 10 years prior to stroke onset.

Subjects underwent standard clinical workup and MRI using a 1.5T whole-body MRI system (Achieva 1.5; Philips Medical Systems, Best, The Netherlands). Upon discharge, subjects were scheduled for outpatient follow-up 3–6 months later, where they were assessed for changes in risk factors, cognition, and function. Cognitive status was assessed by clinical team via structured clinical interview and the Mini-Mental State Examination (MMSE)^18^. If further confirmation was required, the Singaporean version of the Montreal Cognitive Assessment (MoCA)^19^ was conducted. Subjects were classified as having PSCI if they had an MRI-confirmed infarct, met criteria for vascular cognitive impairment^20^, and had MMSE ≤ 25 or MoCA ≤ 22^21^. Remaining subjects were classified as having no cognitive impairment (NCI). Subjects with pre-stroke cognitive impairment, neurological or psychiatric comorbidities, presented outside of the window period, or were unable to undergo cognitive assessments due to severe communication or visual disturbances as a result of the stroke, were excluded. Subjects with significant depression, screened via the 9-point Patient Health Questionnaire (PHQ-9)^22^ were also excluded.

Demographic, clinical, and risk factor data were obtained from admission records. Clinical MR images obtained at the time of stroke were appraised by a neurologist and a neuroradiologist, and visually rated by blinded raters. T1-weighted scans were rated for cortical atrophy using a four-point globalized version of the global cortical atrophy (GCA) score by Pasquier and colleagues^23^, corresponding to none, mild, moderate, and severe cortical atrophy. Acute infarcts were rated on diffusion weighted imaging (DWI) scans with corresponding apparent diffusion coefficient (ADC) maps, and quantified based on number, location and lacunar (3–15 mm largest diameter) vs. non-lacunar (≥15 mm largest diameter). T2-weighted scans, with the corresponding T1 scans for verification, were used to quantify chronic infarcts based on number and location, and to rate severity of white matter hyperintensities (WMH) using the four-point Fazekas scale^24^ signifying none, mild, moderate, and severe WMH. Gradient-echo sequences were used to assess microhemorrhages.

PSCI and NCI subjects were compared for differences in demographics, clinical data, and MR variables using independent sample t-test or Wilcoxon-Mann-Whitney test for continuous data, and χ^2^ test for categorical data. Statistically significant continuous variables were operationalized into categorical variables based on clinically relevant cutoffs^15^ and retested for statistical significance.

### Risk score model building

Variables were deemed eligible for inclusion in the initial stage of model building if they were 1) statistically significant at the univariate level after operationalization, 2) found in the literature to be relevant, and 3) was deemed by the study team that the variables were common enough to be available to existing stroke workflows (see eMethods in Online Supplement for detailed breakdown of literature review and variable selection process). Eligible variables were put into a multivariate binary logistic regression model with PSCI or NCI as the outcome condition. Variables that, in the opinion of the investigators, would not be part of most routine clinical stroke investigations were dropped from analysis, to ensure that the risk score could be easily implemented in existing stroke workflows with no additional assessments or laboratory tests needed. From there, variables were individually dropped from the model if they were not statistically significant and were deemed to be clinically irrelevant given the evidence in existing literature and clinical practice. The logistic model would then be run again to identify the next variable to be dropped. This manual reverse stepwise elimination was carried out until a model was achieved that was overall predictive of PSCI as indicated by the model likelihood ratio (LR) χ^2^ statistic being statistically significant, and contained significant variables with good predictive validity. Based on the study team’s appraisal of the regression model, variables with stronger β coefficients were given point increments of 2 points, while other variables were given one-point increments.

The resultant risk score was tested for predictive accuracy based on discrimination and calibration. Discrimination was assessed via the area under the receiver operating characteristic curve (AUROC). The curve was also used to identify a cutoff point for clinical use that had a balance between sensitivity and specificity, with higher sensitivity being prioritized to reduce the false negative rate. The accuracy, positive predictive value (PPV), and negative predictive value (NPV) of the cutoff were also noted. Prevalence of PSCI was assessed in subjects with low, medium, and high risk on the score, which was defined based on score tertiles. Calibration was assessed by calculating the Brier score and by plotting the scale’s predicted probabilities of PSCI against actual percentages at each point of the scale. Stability was assessed using 10-fold cross validation and appraising the range of variation of the 10 AUROC iterations. Decision curve analysis (DCA)^25^ was performed to compare the clinical utility of the risk score against a “treat all” strategy, “treat none” strategy, and decision making using unweighted age and education cutoffs as clinical risk factors for PSCI.

### Risk score internal validation

Internal validation of the scale was done in a separate cohort of subjects prospectively recruited from the National Neuroscience Institute stroke clinic in Singapore between January 1, 2012 and December 31, 2014, and is similar in design to the development cohort in terms of inclusion criteria and clinical follow-up visits being arranged at 3–6 months post-stroke. A subset of subjects consented to another follow-up at 12–18 months post-stroke for an additional clinical assessment for cognitive status and risk factor progression. Subjects were classified as PSCI or NCI at both the 3–6 months and 12–18 month time-points. Performance of the score, in terms of AUROC, Brier score, 10-fold cross validation, DCA curves, and accuracy statistics of the identified cutoff, was tested at both time-points.

Data imputation was considered in order to manage the loss of cognitive data (i.e. MMSE and MoCA scores at 12–18 months) due to the dropout rate between visits for both the internal and external validation datasets. In both datasets, it was observed that data was likely missing not at random (MNAR) due to the significant differences in 3–6 month PSCI prevalence between subjects that declined follow-up and subjects that presented at 12–18 months (see eTables 1 and 2). Also, variables that would theoretically be associated with post-stroke cognition, and thus likely to be used to impute longitudinal cognitive data, would already be included as factors for the risk score, making it inappropriate to use these factors to impute cognitive outcomes and simultaneously develop a risk score to predict said outcomes. For these reasons, it was determined that multiple imputation and estimation maximization were not appropriate for these datasets.

When considering single imputation methods, the last-observation-carried-forward (LOCF) method was considered as the most suitable method for the current analysis, and 3–6 month cognitive data was carried forward in observations where 12–18 month data was lacking. In general, data imputation via LOCF is not preferable in dementia research due to the false implication that the subjects dropping out do not experience decline or disease progression, thus introducing bias^26^. However, the assumption of minimal or no disease progression is not necessarily violated in PSCI as, unlike the progressive nature of Alzheimer’s disease, PSCI is characterized by cognitive symptoms that are more stepwise in nature^27^. Furthermore, prior evidence looking at post-stroke cognition at 3 months and 15 months post-stroke found that a majority of subjects (76%) experience stable cognitive states and neither progressed not improved between 3 and 15 months^28^. For those reasons, it was determined that LOCF, while not always suitable, was the most appropriate method of data imputation in the current analysis that resulted in the least amount of introduced bias. For clarity, results of risk score performance at 12–18 months will be shown using data undergoing both LOCF imputation and no imputation (i.e. complete case analysis; CCA).

### Risk score external validation

External validation was performed in a third dataset of subjects from the Stroke Registry Investigating Cognitive Decline (STRIDE) study^29^. Subjects were Chinese patients admitted between January 1, 2009 and December 31, 2010 to the acute stroke unit of Prince of Wales Hospital in Hong Kong. Ischemic stroke was identified based on clinical evidence of acute cerebral ischemia with symptoms persisting for at least 24 hours. Exclusion criteria were similar to that of the development and validation cohorts.

Demographic and clinical data were collected during acute hospitalization. MRI was performed for subjects within the first week of stroke admission on a 1.5T (Siemens Sonata, Erlangen, Germany) or a 3.0T (Achieva 3.0; Philips Medical Systems, Best, The Netherlands) whole-body MR system. Three trained neurologists appraised and rated MRI sequences. DWI and ADC sequences were rated for the number and location of small and large acute infarcts. The definitions of small and large infarcts corresponded to that of lacunar and non-lacunar infarcts used in model development. WMH was rated using a globalized four-point version of the age-related white matter changes (ARWMC) scale^30^. Chronic lacunes were identified using fluid-attenuated inversion recovery (FLAIR) sequences verified with T1 scans. GCA was rated in T1 scans using a globalized version of the three-point scale used by Victoroff and colleagues^31^, corresponding to none, mild, and moderate-severe GCA.

Subjects were seen at 3–6 months and 12–18 months post-stroke for cognitive assessment via MMSE, the Hong Kong version of the MoCA, and the Clinical Dementia Rating (CDR) scale^32^. The Geriatric Depression Scale (GDS)^33^ was also used to exclude subjects with cognitive deficits secondary to major depressive symptoms. Subjects were classified as PSCI using the same clinical and cognitive criteria as for internal validation. The risk score was similarly tested for predictive accuracy and utility at both time-points, and data imputation via LOCF was performed for right-censored cognitive data at 12–18 months.

All statistical analysis was done using Stata version 12.0 (StataCorp, College Station, TX, USA). Post-hoc power calculations were done in G*Power Version 3.1.9.2^34^ based on the logistic regression models for the CHANGE score in predicting PSCI outcomes in all 5 scenarios (development, internal validation 3–6 months, internal validation 12–18 months, STRIDE 3–6 months, STRIDE 12–18 months). Univariate statistical analysis and model creation for this study took place from January 4, 2016 to September 30, 2016. Variables were tested for normality of distribution by appraising skewness and kurtosis, and using Shapiro-Wilk test. Significance tests were two-tailed and level of significance was set at p < 0.05, except in stepwise elimination of regression predictors where p < 0.20 was used instead to prioritize model sensitivity. All study procedures were carried out in accordance with institutional guidelines and under approval by the SingHealth Centralised institutional Review Board and the Joint Chinese University of Hong Kong – New Territories East Cluster Clinical Research Ethics Committee. Participants provided written informed consent prior to collection of any research data.

## Results

### Risk score development

1088 individual subjects across three datasets were involved in the creation and validation of the risk score. The development dataset consisted of 243 subjects, of which 34 were excluded (6 presented outside of 3–6 months, 28 had incomplete investigative data). The remaining 209 subjects (32.1% female and 82.8% Chinese) had a mean age of 61.67 years (SD 12.46 years) and a mean education of 4.59 years (SD 4.46 years). At 3–6 months, 78 subjects (37.3%) had PSCI. Age, education, gender, hypertension, atrial fibrillation, GCA, WMH, acute infarcts, chronic lacunes, and intracranial stenosis were found to be significantly different between PSCI and NCI subjects (Table 1) and were included in the initial regression model.

**Table 1:** Univariate analysis of NCI vs. PSCI subjects in the model development dataset.

Variables	NCI N = 131 (62.68%)	PSCI N = 78 (37.32%)	p value
Follow-up interval, mean (SD), days	109.81 (96.34)	124.70 (120.85)	0.830
Age, mean (SD), years	57.34 (11.45)	68.94 (10.61)	<0.001*
Gender, No. (%), female	35 (26.72)	32 (41.03)	0.032*
Education, mean (SD), years	6.35 (4.39)	1.63 (2.67)	<0.001*
Education <6 years, No. (%)	52 (39.69)	67 (85.90)	<0.001*
Race, No. (%)			0.467
Chinese	105 (80.15)	68 (87.18)	
Malay	16 (12.21)	4 (5.13)	
Indian	7 (5.34)	5 (6.41)	
Others	3 (2.30)	1 (1.28)	
Diabetes mellitus, No. (%)	55 (41.98)	31 (39.74)	0.750
Hypertension, No. (%)	94 (71.76)	69 (88.46)	0.005*
Hyperlipidemia, No. (%)	103 (78.63)	66 (84.62)	0.287
Atrial fibrillation, No. (%)	16 (12.21)	17 (21.79)	0.066
Smoking history, No. (%)	45 (34.35)	15 (19.48)	0.006*
History of TIA, No. (%)	12 (9.16)	3 (3.85)	0.150
History of stroke, No. (%)	21 (16.03)	10 (12.82)	0.528
History of IHD, No. (%)	21 (16.03)	19 (24.36)	0.146
GCA score, mean (SD)	0.34 (0.54)	0.56 (0.66)	0.009*
Fazekas WMH score, mean (SD)	1.31 (0.96)	2.01 (0.80)	<0.001^a^
Acute lacunar infarcts, mean (SD)			
Cortical	1.10 (2.21)	1.96 (3.55)	0.326
Sublobar	0.27 (0.69)	0.21 (0.52)	0.404
Infratentorial	0.30 (0.81)	0.37 (1.23)	0.385
Acute non-lacunar infarcts, mean (SD)			
Cortical	0.45 (0.82)	0.85 (1.03)	<0.001*
Sublobar	0.14 (0.39)	0.19 (0.46)	0.363
Infratentorial	0.07 (0.28)	0.04 (0.19)	0.474
Chronic infarcts, mean (SD)			
Cortical	0.29 (0.72)	0.60 (1.00)	0.009*
Sublobar	0.49 (0.92)	0.77 (1.13)	0.022*
Infratentorial	0.21 (0.69)	0.28 (0.66)	0.187

Abbreviations: GCA, global cortical atrophy; IHD, ischemic heart disease; NCI, no cognitive impairment; PSCI, post-stroke cognitive impairment; TIA, transient ischemic attack; WMH, white matter hyperintensity.

*Statistically significant difference (p < 0.05).

From the initial regression model, gender, hypertension, and atrial fibrillation did not achieve significance and were eliminated. The final model included age, education, GCA, acute cortical non-lacunar infarcts, WMH, and chronic lacunes (Table 2), and was overall predictive of PSCI (LR χ^2^ statistic of 73.83; p < 0.001). Age, education, and chronic lacunes were assigned two-point increments, as their β coefficients (0.82, 1.76, and 0.98) were observed to be much stronger than that of GCA, WMH, and cortical infarcts (β = 0.17, 0.24, and 0.31).

**Table 2:** Final multivariate logistic regression model from the model development dataset with PSCI as the outcome variable.

Candidate predictor variables	β coefficient	Standard error	95% CI	p value
Age stages	0.82	0.33	0.17–1.47	0.014*
Education <6 years	1.76	0.43	0.93–2.60	<0.001*
GCA stages	0.17	0.32	−0.45–0.80	0.588
WMH stages	0.24	0.24	−0.22–0.71	0.302
Non-lacunar cortical infarct stages	0.31	0.22	−0.12–0.75	0.158*
Chronic lacunes ≥2	0.98	0.39	0.19–1.73	0.015*
Intercept	−3.53	0.52	−4.54–2.51	<0.001*

Gender, hypertension, and atrial fibrillation were eliminated as insignificant variables.

Abbreviations: CI, confidence interval; GCA, global cortical atrophy; WMH, white matter hyperintensities.

*Statistically significant difference (p < 0.20).

**Figure 1:**
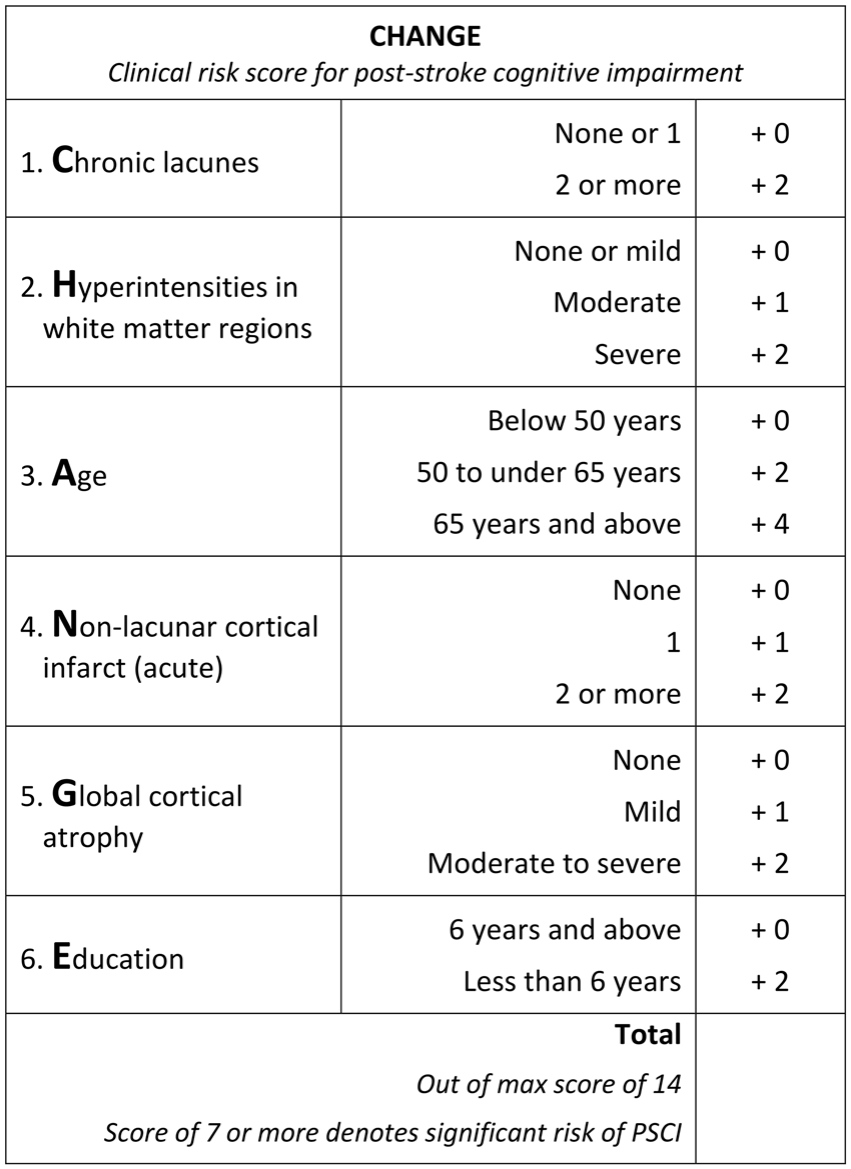
Final CHANGE risk score for clinical use.

**Figure 2:**
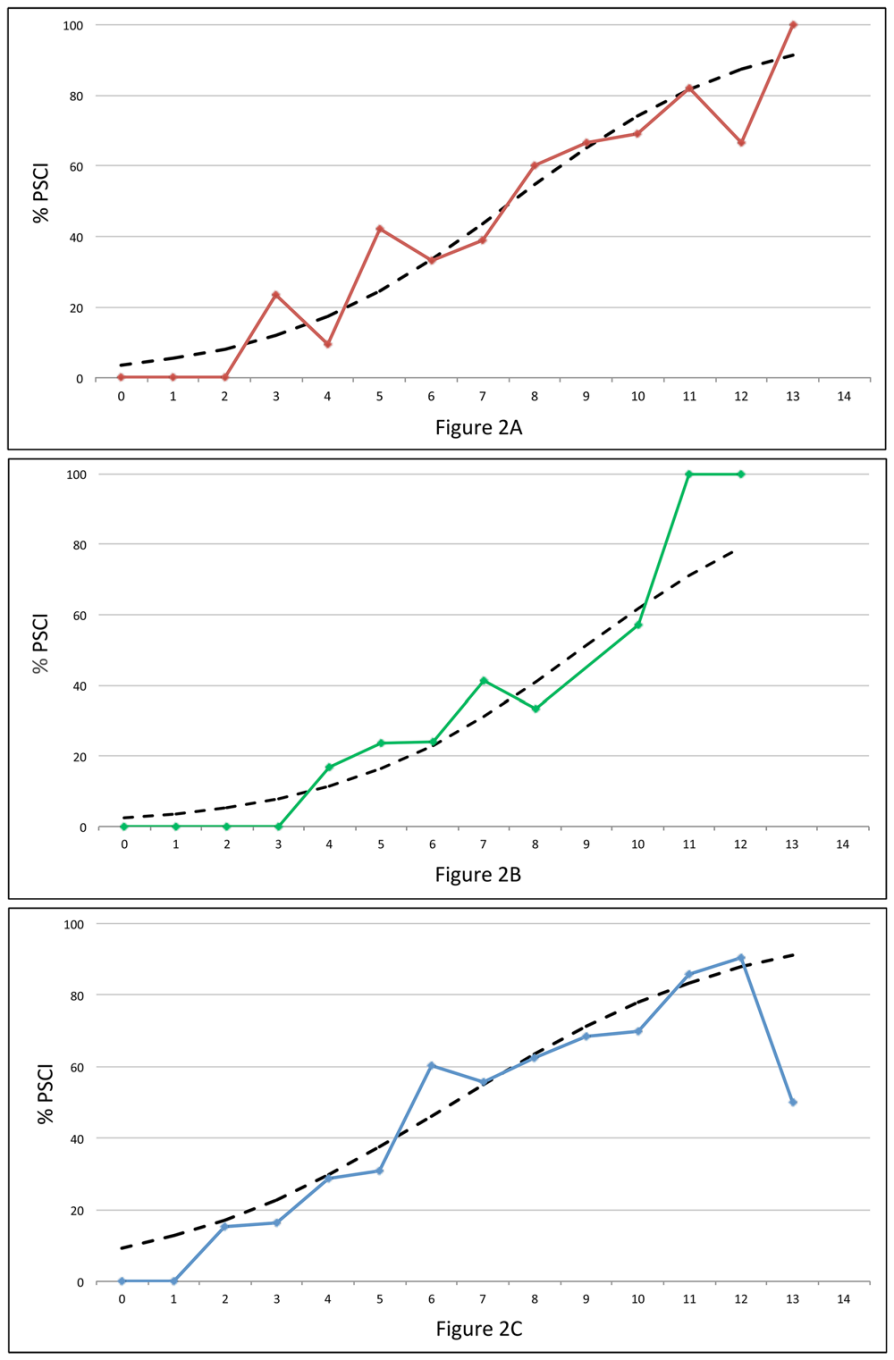
Calibration graphs plotting the predicted probabilities of the risk score (dashed) against the prevalence of PSCI (solid and marked) at each point of the risk score in model development (**A**), internal validation (**B**), and external validation (**C**). There were no observations at score = 14 in all three cohorts, none at score = 9 and 13 in internal validation, and two at score = 13 in external validation.

**Figure 3:**
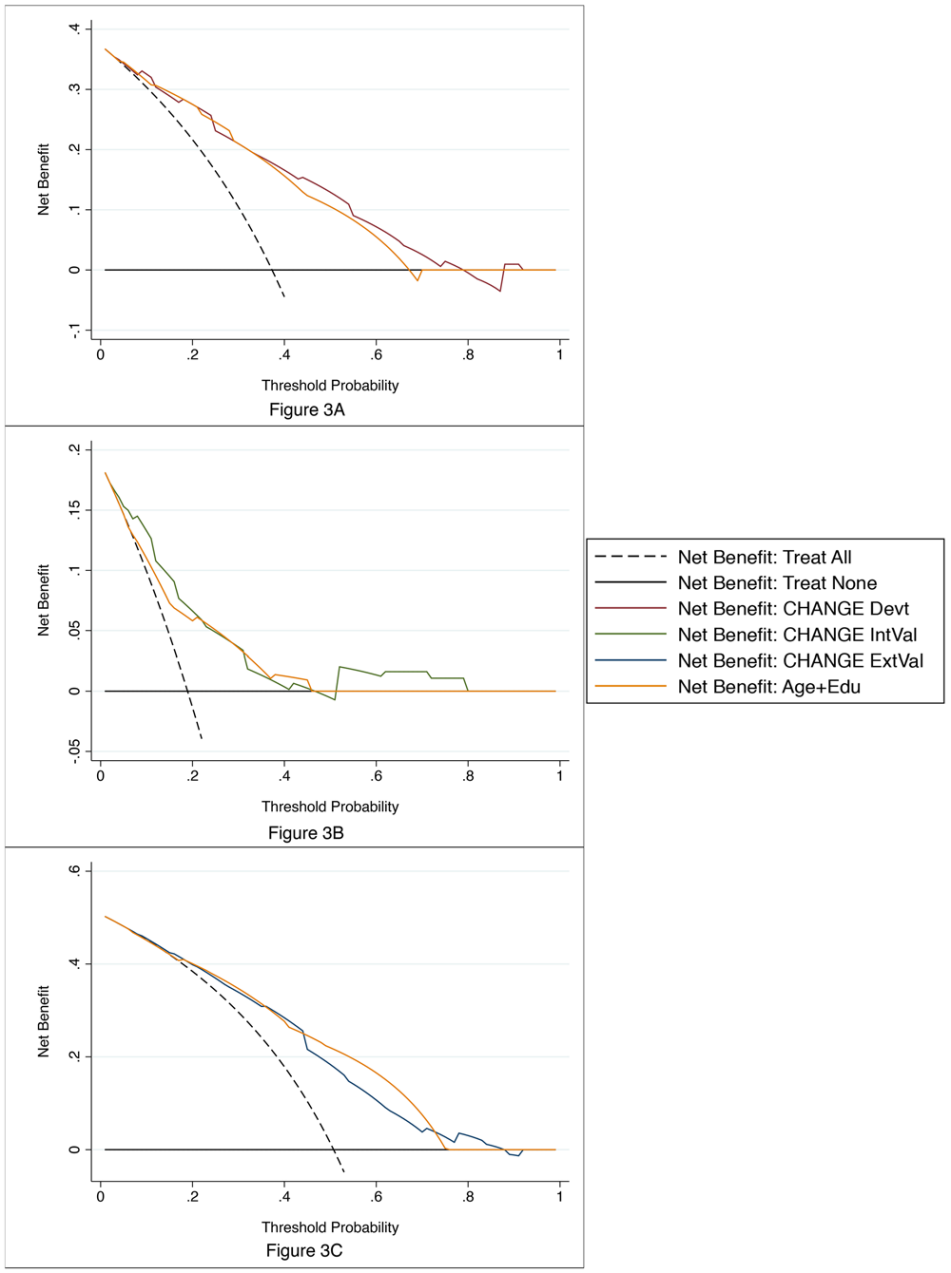
Decision curve analysis demonstrating the utility CHANGE in the development (**A**), internal validation (**B**), and external validation (**C**) cohorts at 3–6 months post-stroke. In each subfigure, the utility of using CHANGE (red, green, blue lines) to screen for PSCI for interventional purposes is compared against the utility of adopting a “treat all” approach (broken black line), a “treat none” approach (solid black line), and an approach to treat based on age and education screening (orange line).

The resultant 0 to 14 point risk score, dubbed CHANGE (Chronic lacunes, Hyperintensities, Age, Non-lacunar cortical infarcts, Global atrophy, and Education; Fig. 1), had an AUROC of 0.82 (95% CI 0.76–0.88). A score of ≥7 was identified as the optimal cutoff, with accuracy of 73.7%, sensitivity of 74.4%, specificity of 73.3%, PPV of 62.4%, and NPV of 83.8%. The score was split into thirds (0–4 points, 5–9 points, and 10–14 points) to evaluate the discriminative ability of the score. 8% of subjects in the lower tertile had PSCI, compared to 49% in the middle tertile, and 75% in the upper tertile. Cross validation yielded AUROC iterations from 0.70 to 0.83 (Table 3). Predicted probabilities plotted against actual proportions of PSCI at each CHANGE point demonstrated that the risk severity for PSCI conferred by CHANGE scores closely followed the actual trends of PSCI prevalence (Fig. 2). DCA curves of CHANGE were better than ‘treat all’ approaches and comparable to age and education cutoffs, with CHANGE outperforming age and education slightly at threshold probabilities between 0.40 and 0.80 (Fig. 3). Post-hoc power analysis of the logistic regression found power to be >0.99.

**Table 3:** Model accuracy, stability, and calibration data in all study cohorts.

	Model Development Cohort	Internal Validation Cohort 3–6 months	Internal Validation Cohort 12–18 months	STRIDE Cohort 3–6 months	STRIDE Cohort 12–18 months
Sample size	209	185^†^	89^†^	693^‡^	567^‡^
PSCI subjects, No. (%)	78 (37.3%)	35 (18.9%)	17 (19.1%)	352 (50.8%)	286 (50.4%)
Stroke to follow-up interval, mean (SD), mo	3.66 (3.24)	3.13 (2.38)	16.35 (4.60)	5.09 (1.29)	12.68 (1.37)
AUROC (standard error)	0.82 (0.03)	0.78 (0.04)	0.79 (0.06)	0.75 (0.02)	0.74 (0.02)
95% confidence interval	0.76–0.88	0.71–0.85	0.68–0.90	0.71–0.79	0.70–0.78
Brier score	0.17	0.13	0.13	0.20	0.21
10-fold cross validation range	0.70–0.83	0.76–0.83	0.78–0.83	0.75–0.78	0.75–0.77
% PSCI at:					
Score 0–4, No. (%)	6 (7.6%)	7 (7.1%)	3 (7.0%)	34 (18.6%)	37 (23.4%)
Score 5–9, No. (%)	48 (49.0%)	21 (27.3%)	10 (25.0%)	229 (57.5%)	181 (55.5%)
Score 10–14, No. (%)	24 (75.0%)	7 (70.0%)	4 (66.7%)	89 (79.5%)	68 (81.9%)
At Score ≥ 7:					
Accuracy	73.7%	77.3%	75.3%	67.6%	66.6%
Sensitivity	74.4%	48.6%	52.9%	71.5%	68.4%
Specificity	73.3%	84.0%	80.6%	63.6%	64.8%
Positive predictive value	62.4%	41.5%	39.1%	67.0%	66.4%
Negative predictive value	83.8%	87.5%	87.9%	68.5%	66.9%
Positive likelihood ratio	2.78	3.04	2.72	1.97	1.94
Negative likelihood ratio	0.35	0.61	0.58	0.45	0.49

^†^Data from Singapore prospective cohort; 12–18 month cohorts are subsets of corresponding 3–6 month cohort.

^‡^Data from STRIDE cohort; 12–18 month cohorts are subsets of corresponding 3–6 month cohort.

Abbreviations: AUROC, area under receiver operating characteristics curve; IQR, interquartile range; PSCI, post-stroke cognitive impairment; STRIDE, Stroke Registry Investigating Cognitive Decline study.

### Internal validation

The internal validation cohort consisted of 185 subjects with stroke workup and 3–6 month follow-up data, of which 35 subjects (18.9%) developed PSCI. As in the development cohort subjects, PSCI subjects here tended to be older, less educated, and hypertensive. PSCI subjects in the internal validation cohort also had a greater prevalence of atrial fibrillation (AF) and prior strokes. For neuroimaging variables, PSCI subjects had worse GCA, WMH, and chronic infarcts (eTable 3 in Online Supplement). 89 subjects consented for follow-up at 12–18 months, with 17 (19%) subjects having PSCI. Of these subjects, 80 were stable at NCI or PSCI between time-points, 3 converted from NCI to PSCI, and 6 reverted from PSCI to NCI. Subjects that underwent 12–18 month follow-up were older than those that declined, but the two groups did not differ significantly in other clinical or demographic variables (eTable 1 in Online Supplement).

Logistic regression using the components of CHANGE at the 3–6 month time-point showed age stages [β = 0.67 (95% CI −0.05–1.40); p = 0.068], GCA stages [β = 1.32 (95% CI 0.65–1.99); p < 0.001], and chronic lacunes [β = 0.69 (95% CI −0.07–1.45); p = 0.075] to be statistically significant at the p < 0.2 level. Low education [β = 0.05 (95% CI −1.42–1.51)], WMH stages [β = 0.66 (95% CI −0.15–1.46)], and non-lacunar infarcts [β = −0.18 (95% CI −1.09–0.73)] were not statistically significant. The model remained predictive of PSCI (LR χ^2^ statistic = 41.48; p < 0.001) with an AUROC of 0.78. CHANGE had an accuracy of 77.3% in the main cohort and 75.3% in the 12–18 month subset. Accuracy, specificity, NPV, and cross validation ranges were comparable between the main cohort, the follow-up subset, and the development cohort (Table 3 and Fig. 2). As in the development cohort, score utility in DCA for CHANGE was comparable to age and education in general, and better than age and education at threshold probabilities between 0.5 and 0.8 (Fig. 3). Performance was similar at the 12–18 month time-point after data imputation by LOCF (eTable 4 in Online Supplement). Power was calculated at 0.98 for CHANGE at 3–6 months and >0.99 at 12–18 months.

### Comparison of CHANGE with SIGNAL_2_

The coefficients of the components of CHANGE in logistic regression models with PSCI at 3–6 months as the outcome in the development and internal validation cohorts were compared to that of the original SIGNAL_2_ scale^15^. In the development cohort, intracranial stenosis was significant as a factor in SIGNAL_2_, but without it in CHANGE, non-lacunar cortical infarcts became significant. Otherwise, CHANGE and SIGNAL_2_ did not differ greatly in terms of direction of effect or significance of the component factors, and Brier scores and cross-validation ranges were similar (eTable 5 in Online Supplement).

### External validation

The external validation cohort consisted of 1007 subjects from STRIDE. 314 subjects were excluded (71 with hemorrhagic stroke, 140 with TIA, 86 with non-stroke or unknown etiologies, and 17 with history of intracranial hemorrhage). The remaining 693 subjects had a mean age of 70.52 (SD 11.08) years, and mean education of 5.48 (SD 4.58) years. PSCI subjects (50.8% of the cohort) tended to be older, less educated, and more hypertensive subjects as in the development and internal validation cohorts, had fewer smokers as in the development cohort, and had greater prevalence of AF and prior strokes as in the internal validation cohort. Uniquely, PSCI subjects in STRIDE also had worse stroke severities and a greater prevalence of diabetes mellitus (Table 4). PSCI subjects also had worse GCA and WMH, more acute cortical large infarcts, and more chronic lacunes (Table 4, eTable 6 in Online Supplement).

**Table 4:** Univariate analysis of NCI vs. PSCI subjects in the model external validation cohort from the STRIDE Study.

Variable	NCI N = 341 (49%)	PSCI N = 352 (51%)	p value
Stroke to follow-up interval, mean (SD), mo	5.03 (1.39)	5.14 (1.19)	0.183
Age, mean (SD) [range], y	65.60 (10.22) [37–95]	75.28 (9.73) [37–96]	<0.001*
Gender, No. (%), female	105 (30.8%)	200 (56.8%)	<0.001
Education, median (IQR), y	7.0 (5.0)	3.0 (6.0)	<0.001*
NIHSS score, median (IQR)	3.0 (4.0)	5.0 (5.0)	<0.001*
Diabetes mellitus, No. (%)	106 (31.1%)	154 (42.9%)	0.001*
Hypertension, No. (%)	226 (66.3%)	259 (73.6%)	0.036*
Hyperlipidemia, No (%)	234 (68.6%)	217 (61.7%)	0.054
Atrial fibrillation, No (%)	48 (14.1%)	76 (21.7%)	0.009*
History of smoking, No. (%)	156 (46.3%)	128 (37.1%)	0.015*
History of drinking, No. (%)	50 (15.1%)	19 (5.5%)	<0.001*
History of TIA, No. (%)	10 (3.0%)	6 (1.7%)	0.281
History of stroke, No. (%)	53 (15.5%)	87 (24.7%)	0.003*
History of IHD, No. (%)	28 (8.4%)	43 (12.3%)	0.089
Family history of stroke, No. (%)	11 (3.2%)	4 (1.1%)	0.059
Victoroff global score, median (IQR)^†^	0.0 (1.0)	0.0 (2.0)	0.015*
ARWMC global score, median (IQR)^†^	1.0 (2.0)	2.0 (3.0)	<0.001*
Presence of acute small infarcts, No. (%)^†^			
Cortical	32 (9.4%)	29 (8.2%)	0.595
Sublobar	132 (38.7%)	127 (36.1%)	0.474
Infratentorial	54 (15.8%)	45 (12.8%)	0.251
Presence of acute large infarcts, No. (%)^†^			
Cortical	21 (6.2%)	42 (11.9%)	0.008*
Sublobar	25 (7.3%)	38 (10.8%)	0.113
Infratentorial	16 (4.7%)	13 (3.7%)	0.511
Presence of chronic lacunes, No. (%), total^†^	166 (48.7%)	237 (67.3%)	<0.001*
Presence of microhemorrhages, No. (%), total^†, ‡^	44 (62.9%)	40 (76.9%)	0.097
Scores at 3–6 month follow-up,			
MMSE, median (IQR)^§^	28.0 (2.0)	21.0 (8.0)	—
MoCA, median (IQR)^§^	25.0 (4.0)	15.0 (7.5)	—
GDS, median (IQR)	3.0 (6.0)	5.0 (6.0)	<0.001*
CDR SoB, median (IQR)	0.0 (0.5)	1.0 (3.0)	<0.001*

^*^Significant at the p < 0.05 level.

^†^Refer to Table 2 of Online Supplement for detailed breakdown of frequency and distribution of neuroimaging findings.

^‡^Data only available for 70 NCI and 52 PSCI subjects.

^§^p value from Mann-Whitney-U test not available as these scores were already considered in the clinical classification of PSCI.

Abbreviations: ARWMC, age-related white matter changes scale for white matter hyperintensities; CDR-SoB, Clinical Dementia Rating – Sum of Boxes score; GDS, Geriatric Depression Scale; IHD, ischemic heart disease; IQR, interquartile range; MMSE, Mini-Mental State Examination; MoCA, Montreal Cognitive Assessment; NCI, no cognitive impairment; NIHSS, National Institutes of Health Stroke Scale; PSCI, post-stroke cognitive impairment; TIA, transient ischemic attack.

In logistic regression, age stages [β = 0.99 (95% CI 0.60–1.37); p < 0.001], low education [β = 1.60 (95% CI 1.24–1.96); p < 0.001], WMH stages [β = 0.28 (95% CI 0.05–0.52); p = 0.023], large cortical infarct stages [β = 0.62 (95% CI 0.09–1.15); p = 0.022], and chronic lacunes [β = 0.64 (95% CI 0.26–1.02); p = 0.001] remained significant, while GCA stages [β = −0.09 (95% CI −0.31–0.14)] were not. CHANGE was overall predictive of PSCI (LR χ^2^ statistic = 199.28; p < 0.001) with an AUROC of 0.75, and demonstrated good accuracy and stability in cross validation (Table 3 and Fig. 2).

At 12–18 months, 567 subjects returned for follow-up. These subjects tended to be younger and have lower NIHSS scores than right-censored subjects (eTable 2 in Online Supplement). 40 (7.1%) converted from NCI to PSCI, and 31 (5.5%) reverted from PSCI to NCI. CHANGE showed similar predictive and calibration performance in the STRIDE 3–6 month cohort, STRIDE 12–18 month subcohort, and the development cohort (Table 3 and Fig. 2). 12–18 month findings in the STRIDE cohort remained similar before and after data imputation (eTable 4 in Online Supplement). CHANGE here has the same net benefits as using age and education in general, and CHANGE outperforms age and education at threshold probability of approximately 0.8 onwards (Fig. 3). Power was found to be >0.99 at both 3–6 months and 12–18 months.

## Discussion

We demonstrate CHANGE score to be reliable in screening ischemic stroke survivors for significant risk of developing PSCI at both the subacute and chronic stages of stroke. The performance of CHANGE has been demonstrated in both internal and external cohorts, and shown to be consistently comparable and stable with good utility. Throughout all three datasets and subcohorts, AUROC ranged from 0.74 to 0.82, and accuracy of the cutoff ranged from 66.6% to 77.3%. This was in spite of all three cohorts being comparable and yet exhibiting some differences in risk factor profiles and visual rating scales.

The variables in CHANGE have been corroborated by the existing literature as being important risk factors for PSCI. A 2005 review identified age, education, silent infarcts, global atrophy, and white matter changes as factors conferring higher risk of post-stroke dementia^2^, while a recent study has emphasized the contributions of excessive lacunes and white matter changes in delayed-onset dementia after stroke or TIA^35^. In addition, non-lacunar infarcts are associated with an odds ratio of 2.4 for PSCI^10^, and cortical infarcts have been shown to impair cognition, especially memory performance, in both humans^36^ and rat models^37^. Although prior evidence has listed recurrent strokes as a significant factor in PSCI^8^, this variable as derived from the subjects’ clinical history was not found to be a useful factor for this scale. It is likely that the measure of chronic lacunes as characterized in MRI scans and used in CHANGE would include both old silent infarcts and previous strokes that have since evolved. Thus, chronic lacunes as a measure should be sufficient to encapsulate the risk of both silent and old infarcts into CHANGE.

CHANGE would be useful in identifying stroke inpatients at significant risk for delayed PSCI, especially those that show good functional recovery and would otherwise have been discharged. At-risk patients may then be prioritized for close clinical monitoring or prophylactic interventions with medication, rehabilitation, or both. While clinical trials in PSCI have shown some limited promise, they suffer from issues such as recruiting subjects with heterogeneous risk profiles^38^. CHANGE could optimize screening for these clinical trials by targeting more high-risk subjects.

It is worth highlighting that DCA curves show CHANGE to be comparable in utility to other known and well used risk factors like age and education, with the added benefit that the framework of the CHANGE scoring system makes it more accessible for practical clinical applications. Furthermore, CHANGE tends to render higher net benefits than age and education at higher threshold probabilities. These tend to be scenarios where the benefits of treating PSCI are good or marginal, while the risks associated with over treating subjects falsely positive for PSCI are high or unknown, as would be the case when considering medications or pharmaceutical trials for new investigative drugs. Thus, while the performance, stability, and ease of use of CHANGE would make it suitable for screening for PSCI in general, its use would be even more strongly recommended in higher risk intervention scenarios.

While CHANGE shares some similarities with SIGNAL_2_, one main difference lies in the lack of intracranial stenosis rated via MRA as a scale component. While MRA was available in our stroke center as part of standard clinical work-up, it may not be so in other stroke centers globally, and inclusion of MRA data into CHANGE might affect its applicability in clinical settings. Performance of CHANGE is comparable to SIGNAL_2_. The slight decrease in sensitivity and AUROC in CHANGE compared to SIGNAL_2 _ is likely negligible to its practical application, with CHANGE having the added benefit of being more widely applicable in stroke centers that do not routinely perform MRA or CTA. Another advantage of CHANGE is the use of qualitative descriptions for severity of cerebral atrophy and WMH, in an effort to make CHANGE more accessible to clinical centers that do not perform quantitative assessments. Even though different WMH and GCA scales were used in the validation cohorts, CHANGE still performed similarly as the WMH and GCA scales used translated well to the corresponding qualitative descriptions in CHANGE. Although the use of the rating scales in this study is recommended, other scales for GCA and WMH that could be translated into the qualitative descriptions utilized by CHANGE could be used with likely minimal issues.

The main strengths of this study include the large combined sample size and the external validation of CHANGE in an independent dataset. Limitations include the possible slight loss of data sensitivity with the harmonization of different rating scales and the censored longitudinal data being MNAR. As the study subjects were Asian, the generalizability of CHANGE to non-Asian cohorts will need to be studied. In addition, the IQCODE may not be sensitive to very early or minor changes in cognition. Thus, some subjects in the study may already have had slight cognitive impairments prior to stroke onset, although not significant enough to warrant attention from clinicians or caregivers. Also, it is plausible that patients not included in this study because they defaulted all follow-up visits, including at 3–6 months, were less impaired than the subjects eventually included in the study. This may have slightly inflated the prevalence of PSCI in the current cohorts, though not to a degree that is inconsistent with the current literature.

In summary, CHANGE demonstrated good performance and accuracy in detecting delayed onset PSCI in independent cohorts from different countries. Its applicability at bedside without the need for additional diagnostic tests should allow for easy implementation by current stroke services. Further work is needed to validate CHANGE in more varied stroke cohorts, and to study the potential of CHANGE in identifying post-stroke patients that may benefit more from cognitive therapy.

## Electronic supplementary material

Online Supplement
