# Supplementary material for: Development and validation of a risk score (CHANGE) for cognitive impairment after ischemic stroke
Source: Sci Rep. 2017 Sep 29;7:12441. doi: 10.1038/s41598-017-12755-z (PMC5622067; doi:10.1038/s41598-017-12755-z)
Supplement: Supplementary file 1 — Online Supplement [file 41598_2017_12755_MOESM1_ESM.pdf]

Development and validation of a risk score (CHANGE) for cognitive impairment after ischemic stroke

Russell J Chander, BA,[1,] Bonnie YK Lam, PhD,[2] Xuling Lin, MRCP,[1] Aloysius YT Ng, BA,[1] Adrian PL Wong, PhD,[2] Vincent CT Mok, FRCP,[2] Nagaendran Kandiah, FRCP[1,3,]*

RJC and BYKL are joint first authors
VCTM and NK are joint last authors
* Corresponding author

Online Supplement

eMethods

Literature review and variable selection

PubMed searches were done with no language restrictions using the search terms “post stroke”, “cognitive impairment”, “dementia”, “vascular”, “ischemic stroke”, “risk”, and/or any appropriate corresponding initialisms (e.g. “PSCI”) in order to identify studies published between 1st January 2004 and 5th September 2016 looking at risk factors associated with cognitive impairment after a mild ischemic stroke, and for methods to quantify post-stroke cognitive impairment (PSCI) risk. The first PubMed search was done on 15th November 2013, and the last search was done on 5th September 2016. Articles were not considered if they included hemorrhagic stroke subjects, non-human studies, or PSCI risk was assessed beyond the initial 2 year period from the time of acute stroke. Only one citation validating an explicit risk score for PSCI (the original $SIGNAL_2$ article that formed the basis of this study) was found. After excluding a further 30 articles (20 reviews, one meta-analysis, one report, two clinical trials, five involving nonstandard/novel procedures, and one where cognition was not explicitly tested), 41 original studies were identified.

The most commonly reported risk factors for PSCI, after excluding 14 factors only assessable outside of acute stroke admission, were age (13 mentions), white matter hyperintensity (ten mentions), education (nine mentions), and infarct location (seven mentions), along with up to 36 other unique factors. These identified risk factors were then identified as potential candidate variables for data collection based on availability of data in clinical work-up of subjects in the development cohort. Variables in the development cohort were collected based on the emergency department presenting workup and discharge reports from neurology inpatient care at a restructured hospital in Singapore. Discussions were held amongst the study team, which included neurologists (XL, VM, and NK), and with other consulting physicians (AC, DO, RS) and a biostatistician (PA) on the feasibility and utility of the variables before beginning analysis. It was at this stage that some variables initially thought to be relevant to PSCI were excluded based on utility.

For example, presenting symptoms, expressed both as raw descriptions of symptoms and categorized into meaningful groups (e.g. lacunar signs or cortical signs), was excluded due to potential reliability problems with patient self-report, and how certain symptoms may have fully or partially resolved before initial clinician assessment. In addition, since the presence of cortical signs was meant to be a surrogate marker for the presence of a cortical stroke, something that could be more robustly characterized in neuroimaging, cortical symptoms as a factor were thus deemed unnecessary. Other candidate factors that could not be included here were 1) physical status on discharge as characterized by modified Rankin scale (mRS), as mRS had already been used as an inclusion criterion for the cohort, 2) apolipoprotein E (ApoE) genotyping, as that information was not available, and 3) intracranial atherosclerosis, as it was in the opinions and experience of the clinicians in the study team that MRA/CTA would not be standard practice for acute stroke work-up in many stroke protocols, especially in regions where healthcare costs to the individual are a significant factor. The factors that underwent univariate analysis in the development cohort had the confidence of the study team that, should they be chosen as variables for the risk score, that other clinical stroke pathways globally would be able to replicate the required variables with little change to their current practice or need for additional data collection.

Variables	Declined longitudinal follow-up N = 96	Underwent longitudinal follow-up N = 89	p value
Age, mean (SD), years	55.86 (11.44)	59.60 (11.18)	0.032*
Gender, No. (%), female	29 (30%)	28 (31%)	0.854
Education, mean (SD), years	9.39 (3.64)	9.01 (3.17)	0.423
Race, No. (%), Chinese	74 (77%)	75 (84%)	0.325
Diabetes mellitus, No. (%)	28 (29%)	31 (35%)	0.409
Hypertension, No. (%)	66 (69%)	66 (74%)	0.416
Hyperlipidemia, No. (%)	92 (96%)	89 (100%)	0.052
Atrial fibrillation, No. (%)	12 (13%)	16 (18%)	0.299
History of smoking, No. (%)	42 (44%)	38 (43%)	0.805
History of TIA, No. (%)	1 (1%)	5 (6%)	0.081
History of stroke, No. (%)	13 (14%)	14 (16%)	0.695
History of IHD, No. (%)	0 (0%)	2 (2%)	0.217
PSCI at 3-6 months, No. (%)	15 (16%)	20 (22%)	0.235

eTable 1: Characteristics of subjects with and without longitudinal follow-up data in the internal validation cohort

* Significant at the $p<0.05$ level

Abbreviations: IHD, ischemic heart disease; TIA, transient ischemic attack

Variables	Declined longitudinal follow-up N = 127	Underwent longitudinal follow-up N = 567	p value
Age, mean (SD), years	72.17 (12.12)	70.15 (10.81)	0.011*
Gender, No. (%), female	50 (39.4%)	255 (45.0%)	0.250
Education, mean (SD), years	5.25 (4.25)	5.53 (4.65)	0.738
NIHSS score, mean (SD)	5.87 (5.70)	4.85 (4.84)	0.042*
Diabetes mellitus, No. (%)	51 (48.2%)	207 (36.5%)	0.442
Hypertension, No. (%)	92 (72.44%)	394 (69.5%)	0.512
Hyperlipidemia, No. (%)	81 (63.8%)	371 (65.4%)	0.724
Atrial Fibrillation, No. (%)	29 (23.0%)	95 (16.8%)	0.097
History of smoking, No. (%)	60 (48.0%)	225 (40.3%)	0.116
History of drinking, No. (%)	12 (9.9%)	57 (10.2%)	0.922
History of TIA, No. (%)	1 (0.8%)	15 (2.7%)	0.212
History of stroke, No. (%)	27 (21.3%)	114 (20.1%)	0.770
History of IHD, No. (%)	16 (12.6%)	55 (9.7%)	0.288
Family history of stroke, No. (%)	0 (0.0%)	15 (2.7%)	0.064
PSCI at 3-6 months, No. (%)	77 (60.6%)	275 (48.5%)	0.010*

eTable 2: Characteristics of subjects with and without longitudinal follow-up data in the STRIDE cohort

* Significant at the $p<0.05$ level

Abbreviations: IHD, ischemic heart disease; TIA, transient ischemic attack

**Variables**	**NCI N = 150 (81.08%)**	**PSCI N = 35 (18.92%)**	**p value**
Follow-up Interval, mean (SD), days	100.02 (76.43)	75.32 (48.86)	0.141
Age, mean (SD), years	55.95 (10.82)	64.98 (11.30)	<0.001*
Gender, No. (%), female	47 (31.33)	10 (28.57)	0.750
Education, mean (SD), years	9.46 (3.33)	8.11 (3.63)	0.027*
Race, No. (%)			0.385
Chinese	117 (78.00)	32 (91.43)	
Malay	17 (11.33)	2 (5.71)	
Indian	7 (4.67)	1 (2.86)	
Others	9 (6.00)	0 (0.00)	
Diabetes mellitus, No. (%)	48 (32.00)	11 (31.43)	0.948
Hypertension, No. (%)	100 (66.67)	32 (91.43)	0.004*
Hyperlipidemia, No. (%)	146 (97.33)	35 (100.00)	0.329
Atrial Fibrillation, No. (%)	15 (10.00)	13 (37.14)	<0.001*
Smoking History, No. (%)	67 (44.67)	13 (37.14)	0.418
Hx of TIA, No. (%)	5 (3.36)	1 (2.86)	0.881
Hx of stroke, No. (%)	17 (11.41)	10 (28.57)	0.010*
Hx of IHD, No. (%)	1 (6.67)	1 (14.29)	0.563
Pasquier GCA score, mean (SD)	0.54 (0.58)	0.94 (0.60)	<0.001*
Fazekas WMH score, mean (SD)	1.67 (0.73)	2.37 (0.69)	<0.001*
Acute lacunar infarcts, mean (SD)			
Cortical	1.09 (2.45)	2.20 (3.29)	0.072
Sublobar	0.46 (0.80)	0.51 (0.95)	0.974
Infratentorial	0.39 (0.86)	0.40 (1.12)	0.541
Acute non-lacunar infarcts, mean (SD)			
Cortical	0.23 (0.48)	0.37 (0.55)	0.096
Sublobar	0.09 (0.29)	0.11 (0.32)	0.707
Infratentorial	0.09 (0.33)	0.06 (0.24)	0.725
Chronic infarcts, mean (SD)			
Cortical	0.44 (0.85)	1.09 (2.52)	0.403
Sublobar	0.77 (1.13)	1.49 (1.96)	0.042*
Infratentorial	0.25 (0.54)	0.34 (0.64)	0.411

eTable 3: Univariate analysis of NCI vs. PSCI subjects in the internal validation cohort.

Abbreviations: GCA, global cortical atrophy; IHD, ischemic heart disease; NCI=no cognitive impairment; PSCI, post-stroke cognitive impairment; TIA, transient ischemic attack; WMH, white matter hyperintensity.

* statistically significant difference (p<0.05)

	Internal Validation Cohort CCA	Internal Validation Cohort LOCF	STRIDE Cohort CCA	STRIDE Cohort LOCF
Sample size	89	185	567	693
PSCI subjects, No. (%)	17 (19.1%)	32 (17.3%)	286 (50.4%)	363 (52.3%)
AUROC (standard error)	0.79 (0.06)	0.80 (0.04)	0.74 (0.02)	0.75 (0.02)
95% confidence interval	0.68 – 0.90	0.73 – 0.87	0.70 – 0.78	0.71 – 0.79
Brier score	0.13	0.13	0.21	0.20
10-fold cross validation range	0.78 – 0.83	0.79 – 0.84	0.75 – 0.77	0.69 – 0.78
% PSCI at:				
Score 0-4, No. (%)	3 (7.0%)	5 (5.1%)	37 (23.4%)	41 (22.4%)
Score 5-9, No. (%)	10 (25.0%)	20 (26.0%)	181 (55.5%)	229 (57.5%)
Score 10-14, No. (%)	4 (66.7%)	7 (70.0%)	68 (81.9%)	93 (83.0%)
At Score ≥7:				
Accuracy	75.3%	77.8%	66.6%	68.1%
Sensitivity	52.9%	50.0%	68.4%	71.3%
Specificity	80.6%	83.7%	64.8%	64.7%
Positive predictive value	39.1%	39.0%	66.4%	68.9%
Negative predictive value	87.9%	88.9%	66.9%	67.3%
Positive likelihood ratio	2.72	3.06	1.94	2.02
Negative likelihood ratio	0.58	0.60	0.49	0.44

eTable 4: Model accuracy, stability, and calibration performance of CHANGE at 12-18 months post-stroke following data imputation.

Abbreviations: AUROC, area under receiver operating characteristics curve; CCA, complete case analysis; IQR, interquartile range; LOCF, last observation carried forward; PSCI, post-stroke cognitive impairment; STRIDE, Stroke Registry Investigating Cognitive Decline study

Candidate predictor variables	CHANGE – Development cohort	$SIGNAL_2$ – Development cohort	CHANGE – Internal validation	$SIGNAL_2$ – Internal validation
Logistic regression results, β (SE) [95% CI]				
Age stages	0.82 (0.33) [0.17 – 1.47]*	0.74 (0.33) [0.09 – 1.39]*	0.65 (0.37) [-0.07 – 1.37]*	0.64 (0.37) [-0.09 – 1.36]*
Education <6 years	1.76 (0.43) [0.93 – 2.60]*	1.59 (0.44) [0.73 – 2.45]*	0.07 (0.74) [-1.38 – 1.52]	0.07 (0.73) [-1.36 – 1.51]
GCA stages	0.17 (0.32 [-0.45 – 0.80]	0.20 (0.33) [-0.43 – 0.84]	0.69 (0.40) [-0.09 – 1.47]*	0.71 (0.40) [-0.07 – 1.50]*
WMH stages	0.24 (0.24) [-0.22 – 0.71]	0.25 (0.24) [-0.22 – 0.72]	1.27 (0.34) [0.59 – 1.94]*	1.27 (0.35) [0.59 – 1.95]*
Non-lacunar cortical infarct stages	0.31 (0.22) [-0.12 – 0.75]*	0.26 (0.23) [-0.19 – 0.70]	0.66 (0.41) [-0.15 – 1.46]*	0.61 (0.42) [-0.21 – 1.43]*
Chronic lacunes ≥2	0.98 (0.39) [0.19 – 1.73]*	0.90 (0.38) [-0.09 – 1.39]*	-0.16 (0.47) [-1.08 – 0.75]	-0.18 (0.47) [-1.09 – 0.74]
Intracranial stenosis	-	0.65 (0.38) [-0.09 – 1.39]*	-	0.27 (0.50) [-0.71 – 1.25]
Intercept	-3.53 (0.52) [-4.54 – -2.51]*	-3.65 (0.53) [-4.70 – -2.61]*	-4.18 (0.65) [-5.45 – -2.91]	-4.24 (0.66) [-5.54 – -2.95]
AUROC (SE) [95% CI]	0.82 (0.03) [0.76 – 0.88]	0.83 (0.03) [0.77 – 0.88]	0.78 (0.04) [0.71 – 0.85]	0.78 (0.04) [0.70 – 0.85]
Brier score	0.17	0.16	0.13	0.13
10-fold cross validation range	0.70 – 0.83	0.76 – 0.88	0.76 – 0.83	0.84 – 0.92

eTable 5: Comparison of regression coefficients and model performance for the CHANGE and the $SIGNAL_2$ scales in the development and internal validation cohorts at 3-6 months post-stroke.

Abbreviations: AUROC, area under receiver operating curve; CI, confidence interval; GCA, global cortical atrophy; SE, standard error; WMH, white matter hyperintensities
* – statistically significant difference (p<0.20)

Variable	NCI N = 341 (49%)	PSCI N = 352 (51%)	χ^2 test p value
Victoroff global score, No. (%)			0.030*
0	183 (53.7%)	163 (46.3%)	
1	94 (27.6%)	94 (26.7%)	
2	64 (18.8%)	95 (27.0%)	
ARWMC global score, No. (%)			<0.001*
0	149 (43.7%)	101 (28.8%)	
1	85 (24.9%)	65 (18.5%)	
2	61 (17.9%)	91 (25.9%)	
3	46 (13.5%)	94 (26.8%)	
Acute cortical small infarcts, No. (%)			0.738
None	309 (90.6%)	323 (91.8%)	
1	20 (5.9%)	18 (5.1%)	
2	4 (1.2%)	4 (1.1%)	
3	4 (1.2%)	5 (1.4%)	
≥4 [max]	4 (1.2%) [8]	2 (0.6%) [6]	
Acute sublobar small infarcts, No. (%)			0.497
None	209 (61.3%)	225 (63.9%)	
1	94 (27.6%)	82 (23.3%)	
2	17 (5.0%)	26 (7.4%)	
3	11 (3.2%)	7 (2.0%)	
≥4 [max]	10 (2.9%) [15]	12 (3.4%) [10]	
Acute infratentorial small infarcts, No. (%)			0.474
None	287 (84.2%)	307 (87.2%)	
1	48 (14.1%)	36 (10.2%)	
2	3 (0.9%)	6 (1.7%)	
3	1 (0.3%)	1 (0.3%)	
≥4 [max]	2 (0.6%) [4]	2 (0.6%) [7]	
Acute cortical large infarcts, No. (%)			0.026*
None	320 (93.8%)	310 (88.1%)	
1	18 (5.3%)	38 (10.8%)	
2	3 (0.9%)	4 (1.1%)	
Acute sublobar large infarcts, No. (%)			0.136
None	316 (92.7%)	314 (89.2%)	
1	24 (7.0%)	38 (10.8%)	
2	1 (0.3%)	0 (0.0%)	
Acute infratentorial large infarcts, No. (%)			0.634
None	325 (95.3%)	339 (96.3%)	
1	13 (3.8%)	10 (2.8%)	
2	2 (0.6%)	3 (0.9%)	
3	1 (0.3%)	0 (0.0%)	
Total chronic lacunes, No. (%)			<0.001*
None	175 (51.3%)	115 (32.7%)	
1	53 (15.5%)	56 (15.9%)	
2	50 (14.7%)	46 (13.1%)	
3	21 (6.2%)	46 (13.1%)	
≥4 [max]	42 (12.3%) [14]	89 (25.3%) [24]	
Total microhemorrhages, No. (%)†			0.074

Variable	NCI<br>N = 341 (49%)	PSCI<br>N = 352 (51%)	χ^2 test<br>p value
None	26 (37.1%)	12 (23.1%)	
1	21 (30.0%)	11 (21.2%)	
2	10 (14.3%)	8 (15.4%)	
3	0 (0.0%)	1 (1.9%)	
≥4 [max]	13 (18.6%) [29]	20 (38.5%) [43]	

eTable 6: Breakdown of frequency of neuroimaging findings in NCI and PSCI subjects.

* Significant at the p<0.05 level

† Data for microhemorrhages available only for 70 NCI subjects and 52 PSCI subjects.

Abbreviations: ARWMC, age-related white matter changes scale; NCI, no cognitive impairment; PSCI, post-stroke cognitive impairment
